# Supplementary material for: Accuracy of a New Pulse Oximetry in Detection of Arterial Oxygen Saturation and Heart Rate Measurements: The SOMBRERO Study
Source: Sensors (Basel). 2022 Jul 3;22(13):5031. doi: 10.3390/s22135031 (PMC9269825; doi:10.3390/s22135031)
Supplement: Supplementary file 1 [file sensors-22-05031-s001.zip › Figure S1.pdf]

Certifications and Patents

- **LIFE METER QUALITY SYSTEMS CERTIFICATIONS:**
  UNI-EN ISO 13485:2016 April 2021

- **BROXY M CE MARK:**
  EC Class 2a Electro Medical Device May 2020

- **FDA PRE-SUBMISSION Q201763 (04.11.2020) Review Complete**

- **Patent Applications and Patents**
  - **First Family**
    - GB 2,572,626 B (a granted GB patent)
    - US 2021/0169382 A1 (a pending published patent application in the USA)
    - EP 3,773,207 A1 (a pending published patent application at the European Patent Office)
    - The above US and EP applications derive from WO 2019/193196 A1 (a published international patent application), which claims priority from the GB application from which the granted GB patent derives
  - **Second Family**
    - GB 2,589,553 A1 (a pending published GB patent application)
    - WO 2021/069729 A1 (a pending published international patent application valid in over 154 countries worldwide)

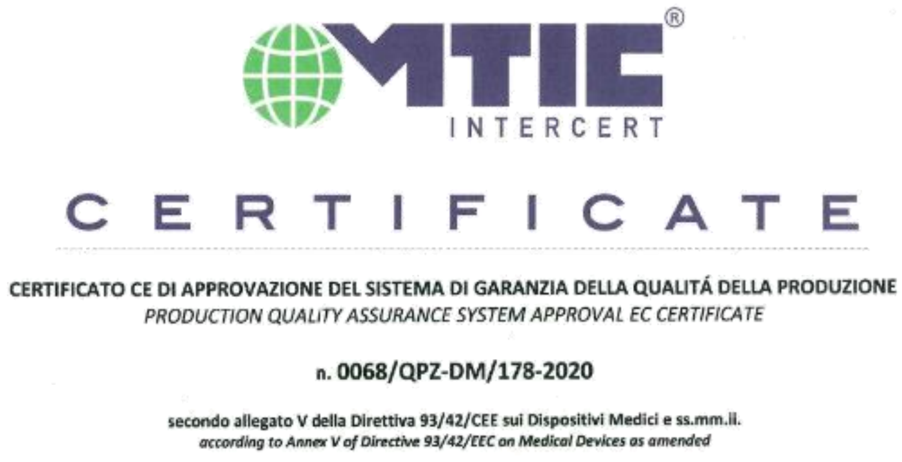
MTIC INTERCERT

CERTIFICATE

CERTIFICATO CE DI APPROVAZIONE DEL SISTEMA DI GARANZIA DELLA QUALITÁ DELLA PRODUZIONE
PRODUCTION QUALITY ASSURANCE SYSTEM APPROVAL EC CERTIFICATE

n. 0068/QPZ-DM/178-2020

secondo allegato V della Direttiva 93/42/CEE sui Dispositivi Medici e ss.mm.ii.
according to Annex V of Directive 93/42/EEC on Medical Devices as amended
